# Association between the T6459C point mutation of the mitochondrial MT‐CO1 gene and susceptibility to sepsis among Chinese Han people

**DOI:** 10.1111/jcmm.13746

**Published:** 2018-09-11

**Authors:** Xiaodong Shen, Guoxin Han, Shuoshuo Li, Yang Song, Hong Shen, Yongzhi Zhai, Yingchan Wang, Fei Zhang, Ning Dong, Tanshi Li, Yongming Yao, Haiyan Zhu

**Affiliations:** ^1^ Emergency Department Chinese PLA General Hospital Beijing China; ^2^ Trauma Research Center First Hospital Affiliated to the Chinese PLA General Hospital Beijing China

**Keywords:** genes, in vitro, inheritance, mitochondria, sepsis

## Abstract

To search for an association between sepsis and mitochondrial genetic basis, we began our study. In this study, a proband harbouring mitochondrial T6459C mutation with sepsis and his Chinese Han pedigree including 7 members of 3 generations were enrolled. General information, blood parameters and mitochondrial full sequence scanning of all members were performed, and cellular functions, including cellular reactive oxygen species (ROS) levels, mitochondrial membrane potential (MMP), degrees of cell apoptosis and adenosine triphosphate (ATP) concentrations, were measured in members with and without the T6459C mutation. Through mitochondrial full sequence scanning and analysis of all members we found, the maternal members (I‐1, II‐1, II‐2 and II‐4) in this Chinese Han pedigree all had the mitochondrial T6459C mutation and were used as the mutation group. The non‐maternal members (II‐3, III‐1 and III‐2) did not have this mutation and were used as the non‐mutation group. The differences in all indicators, including the blood routine, blood biochemistry and coagulation function tests, between members in these two groups were not significant. Under the non‐stimulation condition, the mutation group had higher ROS levels (4210.42 ± 1043.35 vs 3387.78 ± 489.66, *P *=* *.028) and apoptosis ratios (*P *=* *.004) and lower ATP concentrations (*P *=* *.049) and MMP levels (*P *=* *.047) than the non‐mutation group. After 6 hours of simulated LPS stimulation, the mutation group had significantly increased ROS levels (5759.25 ± 2297.90 vs 3862.00 ± 1519.77, *P *=* *.045) compared with the non‐mutation group, whereas the mutation group continued to demonstrate higher ROS levels (*P *=* *.045) and apoptosis ratios (*P *=* *.003) and lower MMP levels (*P *=* *.005) and ATP concentrations (*P *=* *.010). We speculated that the mtDNA T6459C mutation might be the basis for the genetic susceptibility to sepsis.

## 1 INTRODUCTION

With the development of modern medicine and especially the progress in microbiology and immunology, studies on the pathogenic mechanism of sepsis are constantly deepening. The emergence of antibiotics and organ support technology plays important promotion roles in sepsis treatment.^1^ Nevertheless, the sepsis morbidity and mortality rates remain as high as 20%‐30%.^2^ The clinical presentation of sepsis patients is very diverse. Even with the same infection, the occurrence, development and prognosis of the disease differ among patients. An increasing number of scholars have begun to realize that the pathological changes associated with sepsis have obvious individual differences^3^, ^4^ and that different genetic backgrounds were one of the most important factors underlying the individual differences among patients.^5^ This study analysed one Chinese Han pedigree with 7 people in 3 generations to investigate the role of mitochondrial gene mutations in the pathogenesis of sepsis.^6^

## 2 MATERIALS AND METHODS

### 2.1 Study subjects

**Figure 1:**
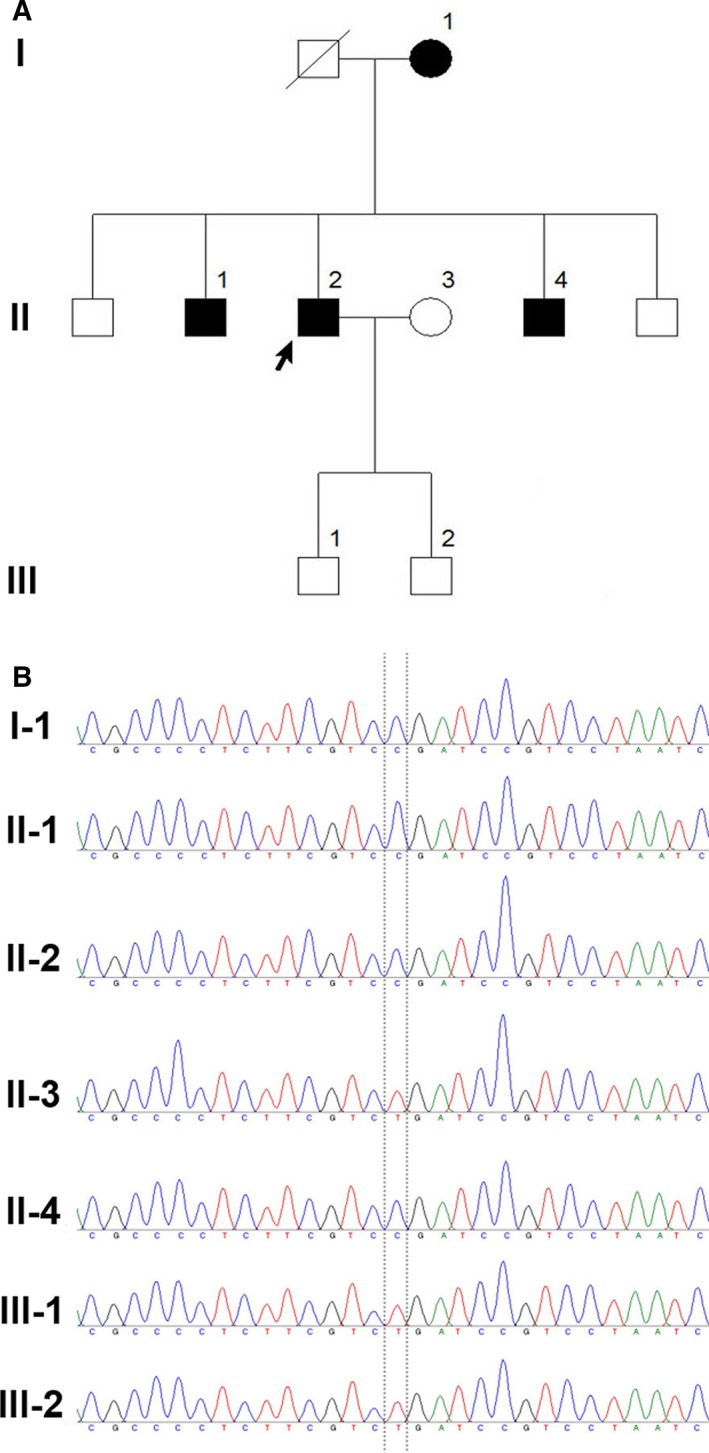
A, The pedigree chart of the 3 generations carrying the mtDNA T6459C mutation for sepsis. B, Schematic diagram of the sequencing results of the mitochondrial 6459 site in the pedigree members

The proband was a 49‐year‐old male from Hebei Province of China (Figure 1A). The patient initially had oral and maxillofacial infections that were followed by pneumonia and a mediastinal infection. The patient had presentations of liver failure and coagulation disorders. And the patient's mother and two brothers were died from sepsis. The diagnosis of sepsis was definite according to the Sepsis‐3 diagnostic criteria.^7^ The maternal inheritance members in the proband's pedigree were I‐1, II‐1, II‐2 and II‐4, and the non‐maternal inheritance members were II‐3, III‐1 and III‐3. This study was approved by the ethics committee of our hospital. All patients signed informed consent forms.

### 2.2 Methods

#### 2.2.1 Recording of general information for the subjects

Age, gender, body height, bodyweight, body mass index (BMI) and past medical history were recorded for all 7 subjects in this pedigree. Additionally, venous blood samples were collected for blood routine, blood biochemistry and coagulation function tests.

#### 2.2.2 Analysis of mitochondrial genes

Mitochondrial DNA in whole blood was extracted using a reagent kit (Promega Wizard, A1120, USA). Primers were designed to amplify all 24 DNA fragments in mitochondria. PCR products were purified using the QIAEXII purification reagent kit (Qiagen). Sequencing and analysis were directly performed using the ABI3700DNA automatic sequencing instrument. Comparison and analysis of DNA sequencing results and corresponding protein sequences were performed using the SeqWeb program GAP (GCG). Comparison of homology was performed using blast from the National Center for Biotechnology Information (NCBI). All sequencing results were compared to the 2013 edition of the Cambridge Reference Sequence (comparison source: MitoMap (http://www.mitomap.org)).

#### 2.2.3 Establishment of lymphocyte cell lines from the subjects

Venous blood samples from the 7 subjects in the pedigree were collected from the median cubital vein using EDTA as the anti‐coagulant. Mononuclear cells from the peripheral venous blood samples were infected with Epstein‐Barr virus (EBV) in this study. B lymphocyte proliferation was induced through the CpG DNA sequence and T lymphocyte proliferation was inhibited using cyclosporine A. Finally, corresponding immortalized B lymphocyte cell lines were established from the subjects.

#### 2.2.4 Detection of cellular reactive oxygen species (ROS) levels

A ROS detection reagent kit (Beyotime) was used. The instruments used were a BD FACSVerse flow cytometer and a Stratos tabletop high‐speed refrigerated centrifuge. 2′,7′‐Dichloro‐dihydro‐fluorescein diacetate (DCFH‐DA) was diluted in serum‐free RPMI 1640 culture medium. After selection of the lipopolysaccharide (LPS) stimulation concentration, the cells were stimulated or not for 6 hours. Then, the cells were washed to remove the DCFH‐DA, resuspended and placed in the flow cytometer for monitoring and recording.

#### 2.2.5 Detection of the mitochondrial membrane potential

An MMP detection reagent kit was used (Beyotime). The instruments used were a BD FACSVerse flow cytometer, Vortex‐5 vortex mixer and Stratos tabletop high‐speed refrigerated centrifuge. After stimulation with the selected LPS concentration, the cell suspension was mixed thoroughly at 0 hour and at the selected time‐points after stimulation. JC‐1 working solution was added, mixed thoroughly and incubated. The cells were centrifuged, washed with JC‐1 buffer and resuspended.

#### 2.2.6 Detection of the cell apoptosis status

A BD Annexin V‐FITC/PI cell apoptosis double staining reagent kit was used. The instruments used were a BD FACSVerse flow cytometer and a Stratos tabletop high‐speed refrigerated centrifuge. The cell lines from the subjects were selected at the logarithmic phase. After non‐stimulation or stimulation with the selected LPS concentration at the selected time points, the cell suspension was mixed thoroughly, and the cells were centrifuged, resuspended, washed, resuspended again and incubated in the dark. Propidium iodide (PI) was added for staining for 5 minutes prior to detection. The binding buffer was used for resuspension. The samples were placed in the flow cytometer for monitoring and recording.

#### 2.2.7 Detection of the cellular adenosine triphosphate concentration

A Promega CellTiter‐Glo Luminescent Cell Viability Assay reagent kit (buffer and substrate lyophilized powder) was used. A BERTHOLD LB 962 CentroLIA/pc luminometer was used. The substrate lyophilized powder was dissolved to obtain the detection reagent. After stimulation with the selected LPS concentration, the cell suspension was mixed thoroughly at 0 hour and at the selected time‐points after stimulation, and the cell density was measured. The samples were added, and control wells were prepared to measure the background luminescence. After the samples were added, they were vortexed in the luminometer and then held still. The luminescence values were measured and recorded.

### 2.3 Statistical methods

All statistical analyses were performed using SPSS 19.0 statistical software. All quantitative data are presented as the mean ± standard deviation and analysed using the *t* test or the Wilcoxon rank sum test. Qualitative data are described using the number of cases and percentages and analysed using the χ^2^ test. *P *<* *.05 was considered statistical significance.

## 3 RESULTS

### 3.1 Analysis of mitochondrial mutations

The maternal inheritance members (I‐1, II‐1, II‐2 and II‐4) in this pedigree all had the mitochondrial T6459C mutation (Figure 1B), and this mutation is highly evolutionarily conserved. These members were used as the mutation group. The non‐maternal inheritance members (II‐3, III‐2 and III‐2) did not have this mutation and were used as the non‐mutation group (Figure 1B).

### 3.2 Baseline level results

Table 1 shows that the differences in all detection indicators between the mutation group and the non‐mutation group do not have significance.

**Table 1:** Comparison of laboratory examination results between the mutation and the non‐mutation groups

Item	Non‐mutation group	Mutation group	*t*	*P*
Haemoglobin (g/L)	151.00 ± 23.81	151.50 ± 16.42	−0.033	.975
WBC count (10 × 9/L)	6.50 ± 2.34	5.89 ± 1.80	0.389	.714
Neutrophil percentage (%)	64.17 ± 3.82	56.18 ± 12.32	1.063	.337
Platelet count (10 × 9/L)	282.33 ± 57.47	209.75 ± 72.42	1.422	.214
CRP (mg/dL)	0.12 ± 0.50	0.20 ± 0.12	−1.136	.307
Alanine aminotransferase (U/L)	17.67 ± 8.62	17.25 ± 6.85	0.072	.946
Aspartate aminotransferase (U/L)	19.00 ± 3.61	21.75 ± 4.72	−0.836	.441
Total serum protein (g/L)	77.33 ± 5.03	74.75 ± 3.30	0.828	.445
Serum albumin (g/L)	48.33 ± 5.03	74.75 ± 3.30	1.682	.153
Total bilirubin (μmol/L)	9.03 ± 2.11	10.38 ± 4.56	−0.465	.661
Direct bilirubin (μmol/L)	3.50 ± 0.62	3.65 ± 1.81	−0.135	.898
Alkaline phosphatase (U/L)	90.67 ± 34.08	62.00 ± 12.30	1.593	.172
γ‐Glutamyl transferase (U/L)	18.00 ± 4.36	27.25 ± 15.39	−1.142	.323
Urea nitrogen (mmol/L)	3.97 ± 0.92	4.53 ± 0.49	−1.048	.343
Serum creatinine (μmol/L)	62.67 ± 13.65	67.15 ± 5.44	−0.625	.560
Blood uric acid (μmol/L)	347.33 ± 120.14	309.25 ± 54.38	0.574	.591
LDH (U/L)	189.33 ± 20.31	218.25 ± 54.02	−0.865	.427
Blood glucose (mmol/L)	4.01 ± 0.83	5.29 ± 1.23	−1.528	.187
Blood potassium (mmol/L)	4.66 ± 0.22	4.75 ± 0.21	−0.546	.608
Blood sodium (mmol/L)	141.33 ± 0.58	141.50 ± 0.58	−0.378	.721
Blood chloride (mmol/L)	102.17 ± 0.15	102.58 ± 0.26	2.371	.064
Thrombin time (s)	16.57 ± 0.90	16.78 ± 0.70	−0.347	.743
APTT (s)	35.20 ± 1.15	13.10 ± 1.77	0.928	.396
Prothrombin time (s)	13.93 ± 0.29	13.30 ± 0.73	1.387	.224
Prothrombin activity (%)	89.33 ± 4.04	99.50 ± 12.50	−1.329	.241
INR	1.07 ± 0.03	1.01 ± 0.73	1.387	.224
Fibrinogen (g/L)	2.21 ± 0.43	2.60 ± 0.41	−1.207	.282

### 3.3 Detection of cellular functions

#### 3.3.1 Detection of ROS levels

**Figure 2:**
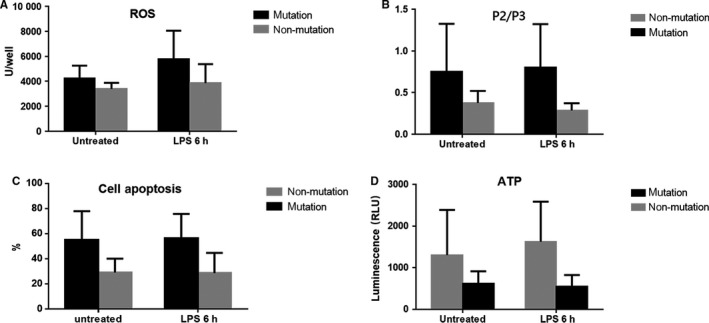
A, Cellular ROS levels in the mutation and non‐mutation groups. B, MMP of the cells in the mutation and non‐mutation groups. C, Cell apoptosis in the mutation and non‐mutation groups. D, Cellular ATP concentrations in the mutation and non‐mutation groups

The detection results are shown in Table 2 and Figure 2A. Under the non‐stimulation condition, the ROS level was significantly higher in the cells from the mutation group than the non‐mutation group (4210.42 ± 1043.35 vs 3387.78 ± 489.66, *P *=* *.028). After 6 hours of LPS stimulation, the ROS level in the mutation group was significantly increased (4210.42 ± 1043.35 vs 5759.25 ± 2297.90, *P *=* *.045), whereas the increasing trend in the non‐mutation group was not significant (3387.78 ± 489.66 vs 3862.00 ± 1519.77, *P *=* *.386). The ROS level in the mutation group was significantly higher than the level in the non‐mutation group (5759.25 ± 2297.90 vs 3862.00 ± 1519.77, *P *=* *.045).

**Table 2:** ROS levels in the cells from the mutation and non‐mutation groups

Group	Mutation group (U/well)	Non‐mutation group (U/well)	*t*	*P*
Untreated	4210.42 ± 1043.35	3387.78 ± 489.66	2.401	.028^a^
LPS 6 h	5759.25 ± 2297.90	3862.00 ± 1519.77	2.143	.045^a^
*t*	−2.126	−0.891		
*P*	.045^a^	.386		

a *P *<* *.05 represent significantly different.

#### 3.3.2 MMP detection

**Figure 3:**
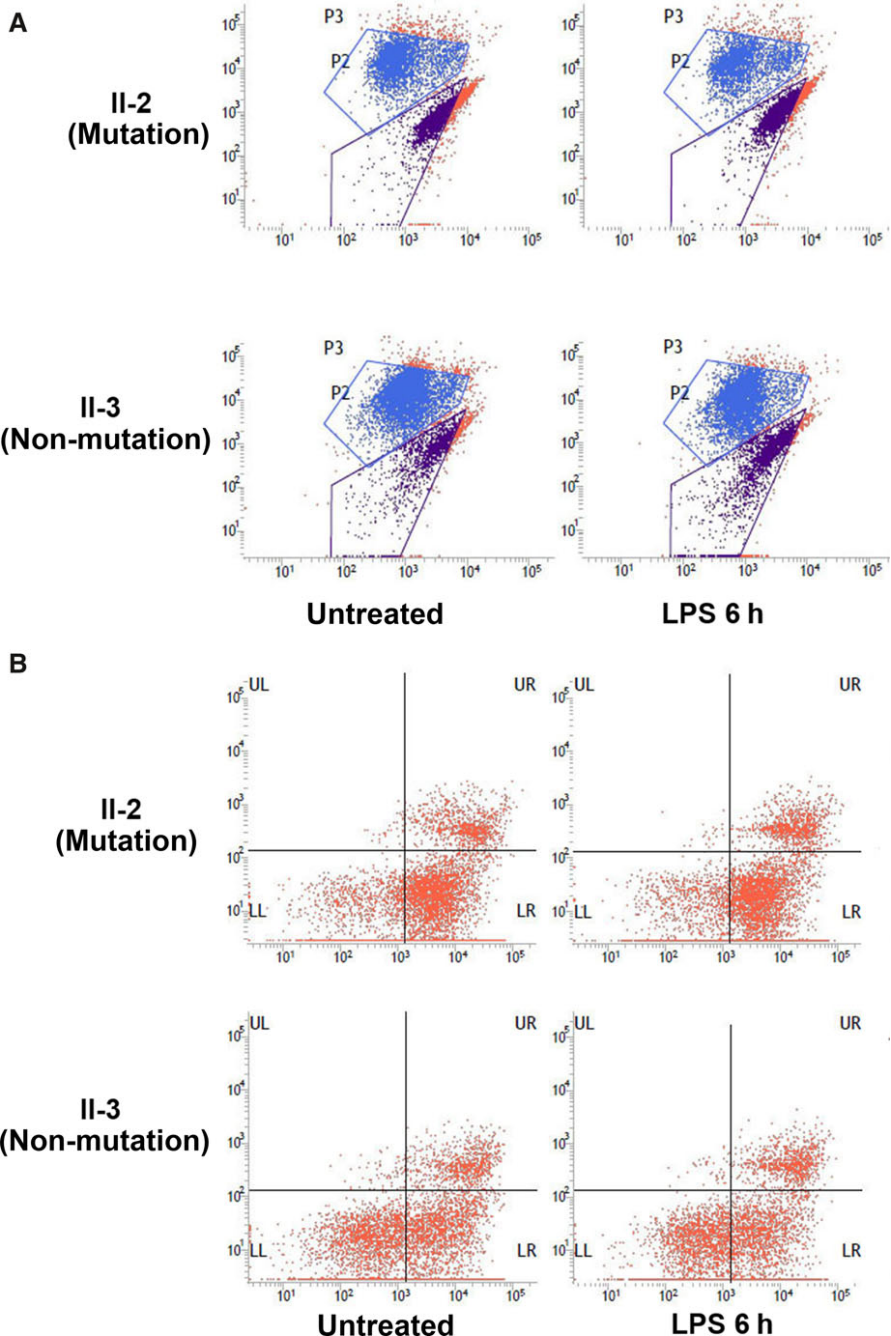
A, Cellular MMP results obtained from the mutation and non‐mutation groups by flow cytometry. B, Cell apoptosis results by flow cytometry in the mutation and non‐mutation groups

The detection results are shown in Figure 3A using II‐2 and II‐3 as examples. The ratio between the JC‐1 monomers and multimers was expressed in the form of P2/P3, which was inversely proportional to the MMP. Under the condition without stimulation, the MMP in the mutation group was significantly lower than the MMP in the non‐mutation group (0.77 ± 0.57 vs 0.38 ± 0.14, *P *=* *.047). After 6 hours of LPS stimulation, the MMP in the mutation group decreased, and the MMP in the non‐mutation group increased. The MMP in the mutation group was significantly lower than the MMP in the non‐mutation group (0.81 ± 0.52 vs 0.29 ± 0.86, *P *=* *.005). Table 3 and Figure 2B.

**Table 3:** MMP of the cells in the mutation and non‐mutation groups

Group	Mutation group	Non‐mutation group	*t*	*P*
Untreated	0.77 ± 0.57	0.38 ± 0.14	2.197	.047^a^
LPS 6 h	0.81 ± 0.52	0.29 ± 0.86	3.412	.005^a^
*t*	0.180	−0.310		
*P*	.859	.761		

a *P *<* *.05 represent significantly different.

#### 3.3.3 Measurement of the cell apoptosis status

The cell lines from all subjects in this pedigree without stimulation or after 6 hours of stimulation with 1 × 10^2 ^ng/mL LPS were loaded into a flow cytometer to detect the cell apoptosis status.^8^, ^9^, ^10^ The detection results are shown in Table 4 and Figure 2C using II‐2 and II‐3 as examples. The number of cells in each region was recorded (Figure 3B). The percentages of cells in the early stage of apoptosis obtained from all cell lines were recorded. And the results in the mutation and non‐mutation groups were compared (54.17 ± 22.76 vs 29.24 ± 10.87, *P *=* *.004). Under the non‐stimulation condition, the apoptosis ratio in the mutation group was significantly higher than the ratio in the non‐mutation group. After LPS stimulation for 6 hours, the apoptosis ratio in the mutation group increased, whereas the apoptosis ratio in the non‐mutation group decreased. The changing trends in these 2 groups were not significant; however, the apoptosis ratio in the mutation group was significantly higher than the ratio in the non‐mutation group (56.32 ± 19.39 vs 28.73 ± 15.94, *P *=* *.003).

**Table 4:** Cell apoptosis in the mutation and non‐mutation groups

Group	Mutation group	Non‐mutation group	*t*	*P*
Untreated	54.17 ± 22.76	29.24 ± 10.87	3.323	.004^a^
LPS 6 h	56.32 ± 19.39	28.73 ± 15.94	3.473	.003^a^
*t*	−0.249	0.079		
*P*	.806	.938		

a *P *<* *.05 represent significantly different.

#### 3.3.4 Detection of cellular ATP concentrations

The detection results are shown in Table 5 and Figure 2D. Without stimulation, the mean value of the intracellular ATP concentration in the mutation group was significantly lower than the mean ATP concentration in the non‐mutation group (620.37 ± 293.09 vs 1304.47 ± 1083.55, *P *=* *.049). After 6 hours of LPS stimulation, the intracellular ATP concentration in the mutation group decreased, and the intracellular ATP concentration in the non‐mutation group increased. The changing trends in these 2 groups were not significant; however, the ATP concentration in the non‐mutation group after stimulation was significantly higher than the ATP concentration in the mutation group.

**Table 5:** Cellular ATP concentrations in the mutation and non‐mutation groups

Group	Mutation group	Non‐mutation group	*t*	*P*
Untreated	620.37 ± 293.09	1304.47 ± 1083.55	−2.103	.049^a^
LPS 6 h	552.93 ± 271.07	1625.28 ± 962.61	−3.247	.010^a^
*t*	0.585	−0.664		
*P*	.564	.516		

a *P *<* *.05 represent significantly different.

## 4 DISCUSSION

As the organelle responsible for oxygen metabolism and energy metabolism in cells, mitochondria are the sites of electron transfer and respiratory movement in the majority of cells. Mitochondria also have functions in the maintenance of calcium balance, cellular signalling pathways and transcription regulation,^11^, ^12^, ^13^ cell growth and cell cycle regulation and are involved in cell apoptosis signalling pathways.^14^ As early as 1962, the association between mitochondria and human diseases drew attention and was studied.^15^ Nowadays, nearly 600 types of mitochondrial gene mutations have been discovered related to diseases.^16^ Sepsis is a disease with genetic factor‐associated susceptibility; the close association between its pathological progression and the structural and functional changes of mitochondria has received attention and been studied.^17^, ^18^ Studies using platelets, which do not contain nuclear genes, as subjects suggested that mitochondrial genes could be used as independent factors to participate in and affect the pathological progression of sepsis.^19^ However, the specific pathogenic mechanism was unknown.

Although the nuclear genes play a major role in genetic regulation, which association with sepsis have been widely studied, the role of mitochondrial DNA cannot be ignored. To make certain of mitochondrial basis of sepsis, we initiated our series studies. The pattern of sepsis development in the Chinese Han pedigree with 7 people in 3 generations included in this study followed the rule of maternal inheritance. The mutation site of the proband was at mtDNA 6459. This site is located on the coding region of the mitochondrial encoded cytochrome c oxidase subunit 1 (MT‐CO1) gene, a functional subunit of the structure of cytochrome C oxidase (COX) at the terminus of the mitochondrial respiratory chain (MRC). COX is also called Complex IV and is the terminal complex of the respiratory chain, accepts and transfers electrons from cytochrome C to oxygen molecules to produce water molecules. Additionally, COX transports 4 protons across the membrane to form the transmembrane proton electrochemical potential difference, necessary for ATP production.^20^, ^21^ The MT‐CO1 gene has a higher degree of conservation during evolution. The discovered T6459C mutation is a non‐synonymous mutation, with the triple base codon changed from UGA to CGA. As a result, the tryptophan in the MT‐CO1 polypeptide chain is mutated into arginine. Tryptophan is a hydrophobic amino acid, and arginine is a basic amino acid; therefore, the secondary structure of the polypeptide might be affected.

The bodyweight, gender and blood parameters (ie, biochemistry) of members in this pedigree were not significantly different (*P *>* *.05). Additionally, the living environment and living habits were similar between the mutation and non‐mutation groups. And the influence of environmental factors on the development of sepsis was considered relatively small. We speculated that the mtDNA T6459C mutation might be involved in the occurrence and development of sepsis. To study the effect of the T6459C mutation on mitochondrial functions, this study infected lymphocytes with EBV to establish cell lines with stable passages.^22^ Additionally, a proper LPS concentration was used to simulate the environment in the cells during the early stage of infection.^8^, ^9^, ^10^ Then, the cellular ROS levels, MMP, apoptosis status and ATP concentrations in the cells were compared.

This study showed that the ROS levels in the mutation group were higher than those in the non‐mutation group before and after LPS stimulation. Additionally, the ROS level in the mutation group was significantly increased after LPS stimulation. Prior to LPS stimulation, the ATP concentration in the mutation group was significantly lower than the ATP concentration in the non‐mutation group. After LPS stimulation, the ATP concentration in the mutation group was significantly decreased, whereas the ATP concentration in the non‐mutation group did not significantly change. Oxygen consumed in the respiratory chain primarily participates in the oxidative phosphorylation reaction of ATP production. Additionally, some oxygen directly interacts with “leaked” electrons from the mitochondrial electron transport chain to form ROS,^23^ primarily in Complex I of the respiratory chain.^24^ However, current increasing evidence has indicated that ROS production by the mitochondria is regulated by immune signalling and plays a role in autoimmunity.^25^, ^26^ The downstream signals of the important inflammatory mediator Toll‐like receptor in the pathological change of sepsis also participate in the production of mitochondrial ROS.^25^ Excessive ROS directly damages mitochondrial proteins to cause membrane structure damage,^27^, ^28^, ^29^, ^30^ such as mitochondrial swelling and the destruction of inner/outer membrane integrity. An excessive inflammatory reaction can also cause down‐regulation of the mitochondrial translation level at the early stage 31, 32 to further cause mitochondrial dysfunction. COX can fix ROS in the reaction during respiration to reduce leakage and free ROS into the cellular environment.^23^ We speculate that the mtDNA T6459C mutation attenuates the fixation function of COX on oxygen while amplifying the production of downstream ROS by Toll‐like receptor signalling, an inflammatory mediator. The T6459C mutation increased ROS production through the above two methods.

ROS is thought to be an important factor that causes mitochondrial injury during the sepsis disease course.^1^ The change in MMP is an important detection indicator reflecting mitochondrial dysfunction. The decrease or disappearance of MMP indicates functional reduction and structural injury of the mitochondria^33^ and reduction or disorder of the ATP production ability. Through the induction of lipid peroxidation, ROS damage mitochondrial structures, induce mitochondrial swelling, and activate the nonspecific permeability transition pore (PTP) on the mitochondrial membrane to cause ion balance disorders, calcium overload, uncoupling of oxidative phosphorylation, and ATP synthesis disorders.^34^

Studies have indicated that mitochondrial injury is usually present at the end stage of sepsis. Early goal‐directed therapy (EGDT) and “Surviving Sepsis Campaign: International Guidelines for Management of Severe Sepsis and Septic Shock”^3^, ^35^ both showed that the first 6 hours of sepsis resuscitation was the key treatment time, especially within 1 hour, which was called “the golden hour” of sepsis treatment. This study stimulated cells with LPS to simulate the internal environment of infection; the early disease course within 6 hour was primarily characterized by a pro‐inflammatory reaction. We observed that cellular functions were decreased, the ATP concentrations and MMP were decreased, and the ROS level and cell apoptosis ratio were increased in the mutation group, suggesting that cells with the mtDNA T6459C mutation could produce significant sepsis‐like pathological changes and exhibit mitochondrial injury. The trend of changes in the ROS levels was most significant after LPS stimulation was introduced into the mutation group. We speculate that the mitochondrial and cellular functional injuries at the early pathological changes associated with sepsis were primarily influenced by ROS. After LPS stimulation, the non‐mutation group exhibited an insignificant ROS increase, showed a slight increase in the cellular ATP concentration and MMP, and demonstrated a slight decrease in the apoptosis ratio; however, all cellular function indicators in the non‐mutation group were significantly different compared to the indicators in the mutation group. These results suggested that cells without the mtDNA T6459C mutation did not produce significant sepsis‐like pathological changes after LPS stimulation.

We speculated that the mtDNA T6459C mutation caused structure and function changes in COX, thereby increasing ROS, cell apoptosis, and genetic susceptibility to sepsis. Additionally, the mtDNA T6459C mutation caused the presence of mitochondrial injury at the early stage of sepsis and induced significant pathological changes associated with sepsis.

## DISCLOSURE

Dr Haiyan Zhu has received grants from Chinese National Natural Science Fund (no.81670467), the Beijing Natural Science Fund (no. 7152136). The remaining authors have disclosed that they do not have any conflicts of interest.
